# The GAP Activity of Type III Effector YopE Triggers Killing of *Yersinia* in Macrophages

**DOI:** 10.1371/journal.ppat.1004346

**Published:** 2014-08-28

**Authors:** Xiaoying Wang, Kaustubh Parashar, Ananya Sitaram, James B. Bliska

**Affiliations:** Department of Molecular Genetics and Microbiology, Center for Infectious Diseases, Stony Brook University, Stony Brook, New York, United States of America; University of California, Davis, United States of America

## Abstract

The mammalian immune system has the ability to discriminate between pathogens and innocuous microbes by detecting conserved molecular patterns. In addition to conserved microbial patterns, the mammalian immune system may recognize distinct pathogen-induced processes through a mechanism which is poorly understood. Previous studies have shown that a type III secretion system (T3SS) in *Yersinia pseudotuberculosis* leads to decreased survival of this bacterium in primary murine macrophages by unknown mechanisms. Here, we use colony forming unit assays and fluorescence microscopy to investigate how the T3SS triggers killing of *Yersinia* in macrophages. We present evidence that *Yersinia* outer protein E (YopE) delivered by the T3SS triggers intracellular killing response against *Yersinia*. YopE mimics eukaryotic GTPase activating proteins (GAPs) and inactivates Rho GTPases in host cells. Unlike wild-type YopE, catalytically dead YopER144A is impaired in restricting *Yersinia* intracellular survival, highlighting that the GAP activity of YopE is detected as a danger signal. Additionally, a second translocated effector, YopT, counteracts the YopE triggered killing effect by decreasing the translocation level of YopE and possibly by competing for the same pool of Rho GTPase targets. Moreover, inactivation of Rho GTPases by *Clostridium difficile* Toxin B mimics the effect of YopE and promotes increased killing of *Yersinia* in macrophages. Using a Rac inhibitor NSC23766 and a Rho inhibitor TAT-C3, we show that macrophages restrict *Yersinia* intracellular survival in response to Rac1 inhibition, but not Rho inhibition. In summary, our findings reveal that primary macrophages sense manipulation of Rho GTPases by *Yersinia* YopE and actively counteract pathogenic infection by restricting intracellular bacterial survival. Our results uncover a new mode of innate immune recognition in response to pathogenic infection.

## Author Summary

The type III secretion system (T3SS) is a macromolecular protein export pathway found in gram-negative bacteria. It delivers bacterial toxins into eukaryotic cells to promote pathogenic infection. T3SSs and the bacterial toxins delivered are critical arsenals for many bacterial pathogens of clinical significance, such as *Yersinia*, *Salmonella* and *Shigella*. On the other hand, the mammalian immune system may recognize the T3SS as a danger signal to signify pathogenic infection, and to stimulate appropriate defense against pathogens. Here, we show that the innate immune system recognizes the activity of YopE delivered by the *Yersinia* T3SS. Modulation of host Rho GTPases by YopE elicits a defensive response, which results in killing of bacteria in host cells. Inhibition of host Rho GTPases by *Clostridium difficile* Toxin B, another bacterial toxin, mimics the YopE-triggered killing effect. Our study demonstrates that host cells sense manipulation of Rho GTPases by bacterial toxins as a surveillance mechanism, revealing new insights into innate immune recognition of pathogenic infections.

## Introduction

Innate immunity provides an early and critical protection against pathogenic infection. In the dominant paradigm of innate immunity, host cells detect pathogens by recognition of “microorganism-associated molecular patterns” (MAMPs) via pattern recognition receptors (PRRs) [1]. However, MAMPs, such as flagellin or lipopolysaccharide (LPS), are conserved microbial structures found in both pathogenic and nonpathogenic bacteria. How then do host cells distinguish pathogens from innocuous microbes? Alternate theories propose that, in addition to MAMPs, host cells also respond to distinct pathogen-induced signals, termed “patterns of pathogenesis” [2]–[4]. Several recent studies have demonstrated that host cells sense the activities of bacterial effectors, such as inhibition of host protein synthesis, activation of host Rho GTPases or pore forming activity, resulting in an active response against the pathogenic attack [5]–[10]. The protective immune response that is triggered by the detection of microbial effectors is defined as an “effector-triggered immune response” (ETIR).

In the genus of *Yersinia*, three species are pathogenic for humans: *Yersinia pestis, Yersinia pseudotuberculosis and Yersinia enterocolitica. Y. pestis* is the causative agent of plague and is typically transmitted by fleabites or aerosols [11], [12]. *Y. pseudotuberculosis and Y. enterocolitica* are associated with self-limiting gastroenteritis acquired from contaminated food or water [11]. The virulence of pathogenic *Yersinia* requires a plasmid (pYV in *Y. pseudotuberculosis*), which encodes a T3SS and a suite of effectors named *Yersinia* outer proteins (Yops) [13]. Upon *Yersinia* infection, Yop effectors are translocated into host cells by the T3SS to modulate host signaling pathways [13]. Four Yop effectors act to target Rho GTPases by distinct mechanisms: YopE mimics the eukaryotic GTPase activating protein (GAP) and promotes GTP hydrolysis to inhibit Rho GTPase activation; YopH, a protein tyrosine phosphatase, impacts Rho GTPase activation by interrupting activating signals for guanine exchange factors (GEFs); YopT, a cysteine protease, proteolytically removes the C-terminal isoprenoid moiety of Rho GTPases, therefore releasing their membrane anchors; YpkA can bind to Rho GTPases with a guanine dissociation inhibitor (GDI) domain [13]. By disturbing Rho GTPase activity, YopE, YopH, YopT and YpkA exert a negative effect on cytoskeleton dynamics, thus contributing to the anti-phagocytic activity of the *Yersinia* T3SS. In addition, YopJ inhibits NF-κB and MAPK signaling pathways, while YopK regulates effector delivery as well as host responses [10]. Translocators YopB and YopD are required for the formation of the T3SS channel and delivery of effector Yops.

The prototypical bacterial effector YopE is a 219 amino acid protein containing a Rho GAP domain (residues 96 to 219) [14]. YopE_GAP_ shares homology with SptP_GAP_ from *Salmonella Typhimurium* and ExoS_GAP_ from *Pseudomonas aeruginosa*. YopE introduces an “arginine finger” into the GTPases catalytic site, which results in efficient GTP hydrolysis and deactivation of GTPases. Exchanging Arg144 in the “arginine finger” with an alanine residue abolishes YopE GAP activity [14]. In mammalian cells, YopE localizes to plasma membrane and unidentified perinuclear compartments, which requires a hydrophobic leucine-rich motif within its membrane localization domain (MLD, residues 53 to 79) [15]–[18]. Stability of YopE in host cells is influenced by allelic variations of residues 62 and 75, as found in different *Yersinia* strains. The presence of lysine residues at position 62 or 75 can mediate YopE ubiquitination and degradation by the host cell proteasome pathway [19]. Both subcellular membrane localization and stability of YopE are important for its GAP activity [16], [19]. YopE is equally effective on Rac1, RhoA and Cdc42 *in vitro* [14], whereas it is preferably active on Rac1 and RhoA, but not Cdc42, *in vivo* [20]. Unlike YopE, YopT seems to be primarily effective on RhoA, but not Rac1 or Cdc42 *in vivo* [21]. However, overexpressed YopT also acts on Rac1 in *Yersinia*-infected epithelial cells [22]. Interestingly, under the latter condition, YopT competes with YopE for the same pool of membrane-associated Rac1, promotes translocation of cleaved Rac1 into the nucleus, and therefore interferes with the ability of YopE to inactivate Rac1 [22].

*Yersinia* is generally considered as an extracellular pathogen, as the bacteria grow primarily in an extracellular form *in vivo*; however, these bacteria can survive and grow inside phagocytic cells, which may be important for the early stages of colonization [23]. It is suggested that macrophages might serve as permissive sites for bacterial replication or even as transport vehicles from the initial site of infection to deeper lymph tissues [24]. Interestingly, T3SS function decreases survival of *Y. pseudotuberculosis* in murine macrophages. Under experimental conditions in which T3SS expression is pre-induced, macrophages restrict intracellular survival of wild-type *Y. pseudotuberculosis*, but not a *yopB^−^* mutant (deficient in Yops translocation) or a *pYV^−^* mutant (missing the entire T3SS) [25]. Thus, some T3SS-dependent factor encoded in the wild-type strain triggers a bactericidal response in macrophages, the mechanism of which remains unclear. It has been shown that upon internalization of *Y. pseudotuberculosis*, the T3SS stimulates Ca^2+^-dependent phagolysosome fusion in macrophages, mediated by the Ca^2+^ sensor SytVII, leading to increased killing of intracellular bacteria [26]. Also, it has been reported that the *Y. pseudotuberculosis* T3SS stimulates Ca^2+^- and caspase-1-dependent lysosome exocytosis, releasing antimicrobial factors [27]. Yet, further studies are needed to determine the molecular basis of innate immune recognition of the *Yersinia* T3SS, and the role of this process in determining the fate of the bacteria in macrophages.

Here we hypothesize that the activities of the *Yersinia* T3SS effectors are sensed by host cells as patterns of pathogenesis, which stimulate an intracellular killing response against *Yersinia* as a type of ETIR. We show that macrophages recognize pathogenic *Y. pseudotuberculosis* through T3SS functions and elicit an intracellular killing response to counteract infection. We provide evidence that YopE GAP activity is a critical factor sensed by macrophages, with YopH playing a minor role. Overexpression of YopT counteracts the YopE-triggered killing effect possibly by competing for the Rho GTPase target and by reducing YopE translocation. Also, this YopE-triggered intracellular killing response can be mimicked by other bacterial derived toxins like *Clostridium difficile* Toxin B, indicating that host cells sense manipulation of Rho GTPases as a conserved surveillance pathway to detect pathogens. Thus, our data provide another example of a protective host response induced by pathogenic bacteria through recognition of bacterial effector activities on Rho GTPases, revealing a novel mode of innate immune recognition towards pathogenic infection.

## Results

### YopE and YopH restrict survival of *Yersinia* inside macrophages

**Figure 1:**
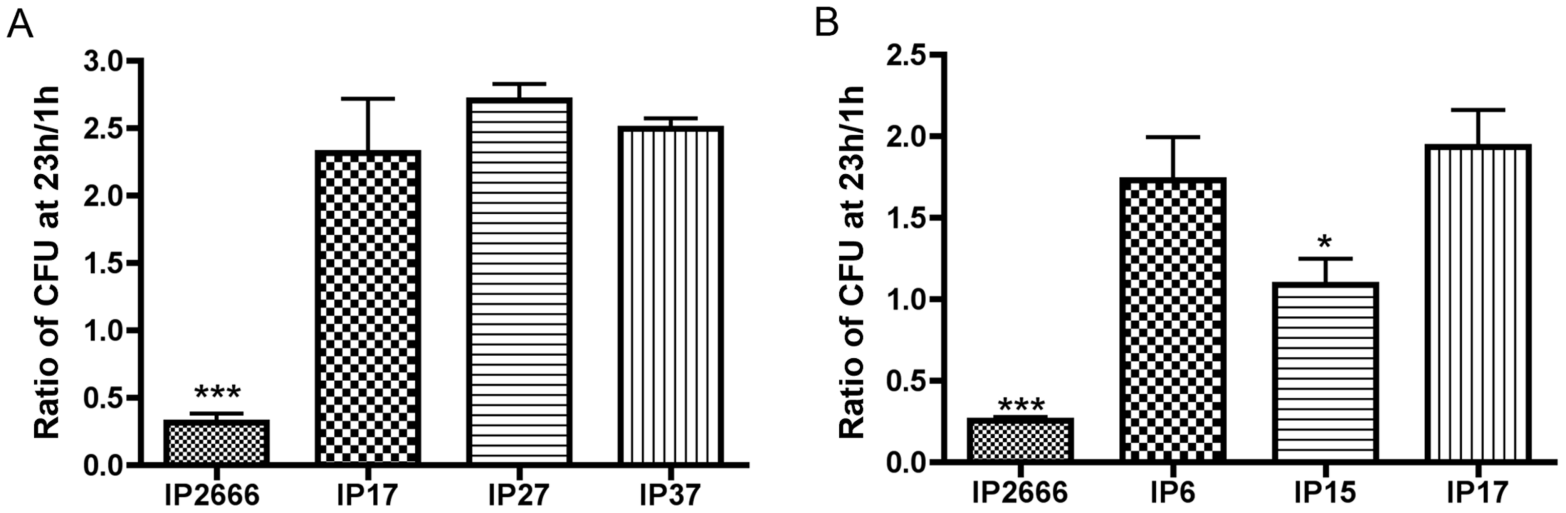
Comparison of different *Y. pseudotuberculosis* strains for survival inside macrophages as determined by CFU assay. BMDMs were infected with the indicated strains at an MOI of 10 for 20 min, followed by gentamicin treatment to eliminate extracellular bacteria. At 1 h and 23 h post infection, the infected BMDMs were lysed, and serial dilutions were plated to determine the survival of intracellular bacteria by CFU assay. Ratios of CFU at 23 h/1 h are shown, determined as [23 h post infection CFU/1 h post infection CFU]. Results shown are the means from three independent experiments with duplicate infection wells. Error bars show standard deviations. (A)***, P<0.001 compared to IP17; (B) *, P<0.05 and ***, P<0.001 compared to IP6, as determined by one-way ANOVA.

Previous studies have shown that T3SS decreases survival of *Y. pseudotuberculosis* in murine macrophages [25], [26]. To determine if specific Yop effectors might contribute to decreased survival of *Y. pseudotuberculosis* in macrophages, the wild-type strain IP2666 and several *yop* deletion mutants were studied. Initially, the survival of IP2666 (wild-type), IP17 (*yopEH^−^*), IP27 (*yopEHJ^−^*) and IP37 (*yopEHJMKYpkA^−^*) (Table 1) in murine bone marrow-derived macrophages (BMDMs) was compared. Naïve BMDMs were infected with the indicated strains, followed by gentamicin treatment to eliminate extracellular bacteria. At 1 h and 23 h post infection, infected BMDMs were lysed and spread on LB plates to enumerate viable bacteria. CFU at 1 h post infection was considered as the initial intracellular bacterial count. The ratio of CFU between 23 h and 1 h post infection was calculated for each strain. At 1 h post infection, IP2666 showed lower CFU as compared to IP17, IP27 and IP37 (Figure S1A). This is expected because IP2666 expresses Yops with anti-phagocytic functions (YopE and YopH); however the other strains are *yopEH^−^* mutants. At 23 h post infection, IP17 displayed significantly higher level of CFU as compared to IP2666, but similar level as compared to IP27 and IP37 (Figure S1B). Consistently, for the ratio of CFU at 23 h/1 h, the level of IP17 was significantly higher than IP2666, but similar to IP27 and IP37 (Figure 1A). To rule out a threshold effect due to the differences in the initial bacterial uptake, BMDMs were infected with IP2666 or IP17 at different MOIs (Figure S2). Even with higher CFU at 1 h post infection, IP2666 (MOI = 10) still showed decreased survival in comparison to IP17 (MOI = 5 or 2.5) at 23 h post infection (Figure S2AB). Therefore, IP2666 shows reduced intracellular survival, in contrast to IPI7, which shows an intracellular growth phenotype, indicating that deletion of *yopE* and *yopH* promotes *Yersinia* survival inside macrophages.

**Table 1:** *Yersinia pseudotuberculosis* strains used in this study.

Strain or plasmid	Relevant Characteristics	Reference
IP2666	Wild-type, pYV^+^, naturally *yopT^−^*	[60]
IP6	*yopE^−^*	[14]
IP15	*yopH^−^*	[61]
IP17	*yopEH^−^*	[61]
IP27	*yopEHJ^−^*	[62]
IP37	*yopEHJMKypkA^−^*	[63]
IP40	*yopB^−^*	[64]
IP2666+empty vector	IP2666 (pMMB67HE)	This study
IP6+empty vector	IP6 (pMMB67HE), *tac* promoter	[14]
IP37+empty vector	IP37 (pMMB67HE)	This study
IP6+pYopE	IP6 (pYopE), *yopE* controlled by *tac* promoter	[14]
IP6+pYopER144A	IP6 (pYopER144A)	[14]
IP6+YopE	IP6 (pPEYopE), *yopE* controlled by native promoter	[30]
IP6+YopE3N	IP6 (pPEYopE L55N I59N L63N)	This study
IP6+YopER62K	IP6 (pPEYopER62K)	This study
IP6+YopEL109A	IP6 (pPEYopEL109A)	This study
IP6+YopE-SptP	IP6 (pPEYopE_1–100_ SptP_166–293_)	This study
IP2666+YopT	IP2666 (pPTYopT), *yopT* controlled by native promoter	[30]
IP6+YopT	IP6 (pPTYopT)	[30]
IP2666+pYopT	IP2666 (pYopT), *yopT* controlled by *yopH* promoter	[30]
IP2666+pYopTC139S	IP2666 (pYopTC139S)	This study
IP6+pYopT	IP6 (pYopT)	This study
IP6+pYopTC139S	IP6 (pYopTC139S)	This study
IP37+pYopT	IP37 (pYopT)	This study
IP37+pYopTC139S	IP37 (pYopTC139S)	This study
IP37+pYopTH258A	IP37 (pYopTH258A)	This study

**Figure 2:**
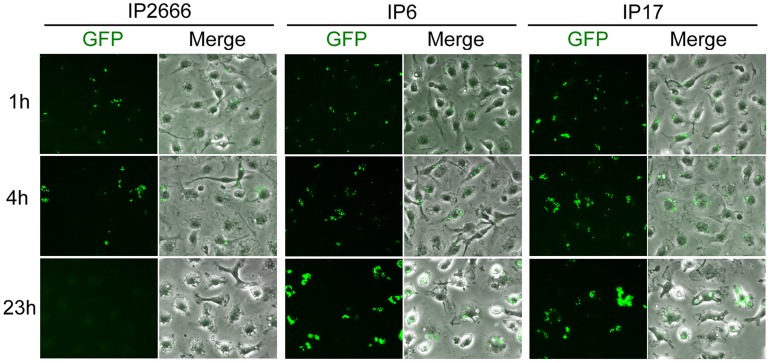
Comparison of different *Y. pseudotuberculosis* strains for survival inside macrophages as determined by fluorescence microcopy. BMDMs were infected with the indicated GFP-encoding strains at an MOI of 10, as described in Figure 1. At 1 h, 4 h and 23 h post infection, the infected BMDMs were fixed and analyzed by fluorescence microscopy. One hour before fixation, IPTG was added to induce de novo expression of GFP from viable intracellular bacteria. Shown are GFP or an overlay of GFP and phase contrast signal from representative images.

To further elucidate the effects of YopE and YopH on intracellular survival of *Yersinia*, IP2666 (wild-type), IP6 (*yopE^−^*), IP15 (*yopH^−^*) and IP17 (*yopEH^−^*) (Table 1) were compared by CFU assay (Figure 1B). IP6 showed an intracellular growth phenotype similar to IP17, while IP15 had an intermediate phenotype (Figure 1B). The results were further confirmed by fluorescence microscopy. IP2666, IP6 and IP17 encoding GFP were used to infect BMDMs for different lengths of time. One hour before fixation and examination of the samples by fluorescence microscopy, IPTG was added to induce de novo expression of GFP from viable intracellular bacteria (Figure 2). At 23 h post infection, IP6 and IP17 showed greater survival compared to IP2666 (Figure 2). These results demonstrate that YopE is required for reduced survival of *Yersinia* in macrophages, and YopH cooperates with YopE in this process.

To determine whether SytVII-mediated phagolysosome fusion contributes to YopE-dependent intracellular killing, *SytVII^−/−^* BMDMs were compared to wild-type BMDMs for their ability to restrict intracellular survival of IP2666, IP17 or IP40 (*yopB* mutant, Table 1). The *SytVII^−/−^* genotype was verified by PCR using mouse-tail genomic DNA, in comparison to wild-type mice (Figure S3A). No significant difference was observed by CFU assay for IP2666 survival inside wild-type or *SytVII^−/−^* BMDMs (Figure S3B), suggesting that SytVII-mediated phagolysosome fusion does not contribute to the YopE-dependent killing of *Yersinia* in macrophages.

Under our experimental conditions, *Yersinia* infection does not cause significant cell death of macrophages (below 2% LDH release after 23 h from IP2666 or IP6 infected macrophages, data not shown). Accordingly, reduced intracellular survival of IP2666 is not due to enhanced *Yersinia*-induced macrophage cell death. We also investigated the possibility that IP2666 infection induces gentamicin uptake and leads to enhanced bacterial killing by gentamicin. If this is true, with increasing amount of gentamicin, intracellular IP2666 would be more sensitive than IP17, due to more gentamicin uptake. To analyze this possibility, the survival of IP2666 and IP17 in macrophages was compared with increasing amount of gentamicin. IP2666 and IP17 responded similarly to increasing amounts of gentamicin (Figure S4), indicating that reduced intracellular survival of IP2666 is not due to increased gentamicin internalization.

### GAP activity of YopE is essential for reduced survival of *Yersinia* in macrophages

**Figure 3:**
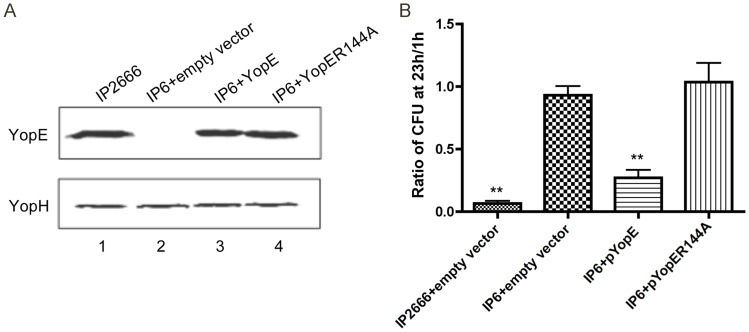
Measurement of YopE production and survival in macrophages by different *Y. pseudotuberculosis* strains. (A) Static expression levels of YopE in the indicated strains as determined by immunoblotting analysis. Indicated bacteria strains were grown at 37°C in the presence of 2.5 mM CaCl_2_ for 2 h. Bacterial pellets were collected and analyzed by immunoblotting using antibodies specific for YopE. YopH levels are shown as loading controls. (B) Survival of intracellular bacteria was determined by CFU assay in infected macrophages (MOI of 10), as described in Figure 1. Results shown are the means from three independent experiments with duplicate infection wells. Error bars show standard deviations. **, P<0.01 compared to IP6+empty vector, as determined by one-way ANOVA.

To investigate if the GAP activity of YopE is crucial for macrophages to restrict survival of intracellular *Yersinia*, experiments were carried out to compare survival of bacteria producing YopE or YopER144A. In YopER144A, a single substitution of arginine to alanine was introduced at amino acid 144 to yield a catalytically dead protein [14]. A plasmid vector encoding *yopE* or *yopER144A* was introduced into IP6 (Table 1). The production level of YopE or YopER144A from the vector in trans was similar to the native level in the wild-type strain as shown by SDS-PAGE and immunoblotting (Figure 3A, compare lanes 1, 3 and 4). The survival of IP6+pYopE and IP6+pYopER144A in macrophages was then compared. IP2666 or IP6 containing the empty vector (Table 1) were analyzed in parallel as controls. IP6+pYopE displayed a reduced intracellular survival phenotype, similar to IP2666+empty vector (Figure 3B). In contrast, IP6+pYopER144A showed increased intracellular survival, comparable to IP6+empty vector (Figure 3B). Unexpectedly, the empty vector (pMMB67HE) had a negative effect on *Yersinia* survival inside macrophages (Figure S5), possibly due to the metabolic burden introduced by the plasmid [28], [29]. Nevertheless, these results demonstrate that YopE GAP activity is indispensable for causing reduced survival of *Yersinia* in macrophages, and we hypothesize that YopE GAP activity is somehow recognized by macrophages, triggering increased killing of intracellular *Yersinia*.

### Overexpression of YopT counteracts YopE-triggered killing of *Yersinia* in macrophages

**Figure 4:**
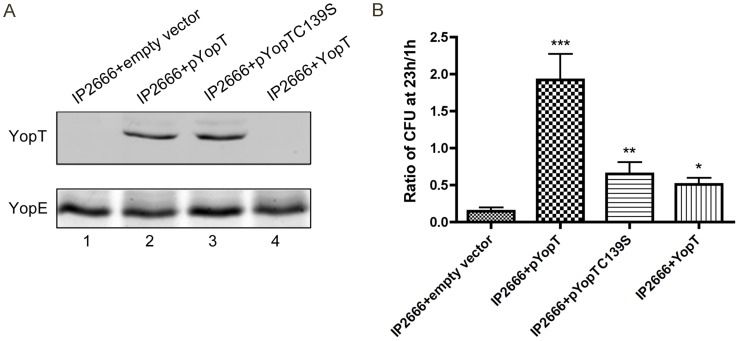
Measurement of YopT production and survival in macrophages by different *Y. pseudotuberculosis* strains. (A) Amounts of YopT secreted by different strains as determined by immunoblotting analysis. Indicated strains were grown at 37°C for 4 hours under low Ca^2+^ conditions. TCA-precipitated supernatants were collected and analyzed using antibodies specific for YopT (upper panel). YopE levels as determined by staining with GelCode Blue Stain Reagent (bottom panel). (B) Intracellular bacterial survival was determined by CFU assay in infected macrophages (MOI of 10), as described in Figure 1. Results shown are the means from four independent experiments with duplicate infection wells. Error bars show standard deviations. ***, P<0.001, **, P<0.01 and *, P<0.05 compared to IP2666+empty vector, as determined by one-way ANOVA.

Given the activity of YopT towards Rho GTPases and its crosstalk with YopE, the potential influence of YopT on survival of *Yersinia* inside macrophages was studied. IP2666 is a *yopT* mutant due to a naturally-occurring deletion in pYV in this strain [30]. Plasmids that overexpress YopT or catalytically-inactive YopTC139S were introduced into IP2666; control strains containing the empty vector or a plasmid producing native levels of YopT under its native promoter were also constructed (Table 1). Analysis of proteins secreted by the bacteria under low calcium conditions using SDS-PAGE and immunoblotting showed that YopT and YopTC139S were overproduced at equal levels, while the native level of YopT was undetectable (Figure 4A, compare lanes 2, 3 and 4). Interestingly, when these strains were used to infect macrophages, overexpression of YopT in IP2666 significantly increased *Yersinia* intracellular survival, giving the opposite effect of YopE (Figure 4B and Figure S6A). *Yersinia* survival in macrophages was moderately increased when YopTC139S was overexpressed in IP2666 (Figure 4B and Figure S6A), indicating that YopT catalytic activity is important for counteracting the YopE-triggered killing effect. Expression of YopT at native level in IP2666 also slightly improved *Yersinia* intracellular survival (Figure 4B and Figure S6A).

Using detergent extraction assay and immunoblotting, lysates of infected macrophage were analyzed to detect the amounts of YopE that were translocated by the different strains. Overexpression of YopT or YopTC139S in IP2666 diminished YopE translocation to 8% or 25% of wild-type level respectively (Figure S7AB). Native level of YopT in IP2666 slightly reduced YopE translocation (75% of wild-type level) (Figure S7AB).

Active and inactive YopT proteins were overexpressed in IP6 or IP37 to further examine the mechanism by which this effector counteracts killing of *Yersinia* in macrophages. Overexpression of YopT or YopTC139S in IP6 equally enhanced bacterial survival (Figure S6B), while overexpression of active or inactive YopT proteins in IP37 had no effect on *Yersinia* survival inside macrophages (Figure S6C).

Taken together, these results suggest that YopT has the ability to counteract YopE-triggered intracellular killing effect, which is partially dependent on YopT protease activity. YopT catalytic activity may counteract the YopE effect by competing with YopE for a Rho GTPase target or by reducing YopE translocation. Thus, inactivation of a Rho GTPase by a specific mechanism, i.e. GAP mechanism, appears to be sensed by macrophages, resulting in increased killing of intracellular *Yersinia*.

### Membrane localization, stability and target specificity of YopE are important for killing of *Yersinia* in macrophages

**Figure 5:**
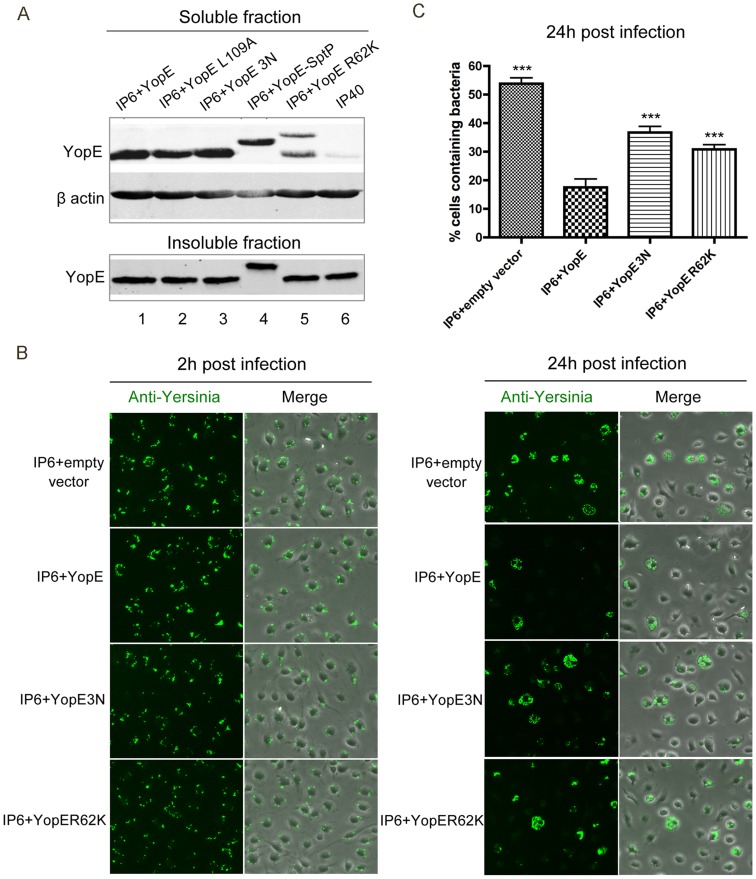
Measurement of YopE translocation and survival in macrophages by different *Y. pseudotuberculosis* strains. (A) YopE translocation levels in BMDMs infected by the indicated strains, determined by detergent extraction assay and immunoblotting analysis. BMDMs in 6 well plates were infected at an MOI of 30 for 2 h, then lysed using 1% Triton X-100 buffer. Cell lysates were centrifuged to obtain soluble and insoluble fractions, which were subjected to immunoblotting analysis using antibodies specific for YopE. β-actin levels from the soluble fraction are shown as loading controls. (B) BMDMs were infected with the indicated strains at an MOI of 10, as described in Figure 1. At 2 h and 24 h post infection, infected cells were fixed, permeabilized and labeled with a rabbit anti-*Yersinia* antibody (green). Shown is bacteria or an overlay of bacteria and phase contrast signal from representative images obtained by fluorescence microscopy. (C) Percentage of macrophages containing fluorescent *Yersinia* at 24 h post infection, quantified from three independent microscopic experiments as described in (B). Error bars show standard deviations. ***, P<0.001 compared to IP6+YopE, determined by one-way ANOVA.

Localization to membranes and stability of YopE are critical for functional GAP activity against Rho GTPases in host cells [15], [16], [19]. Since our results suggest that YopE GAP activity is sensed by macrophages, we hypothesize that membrane localization and stability of YopE will impact its ability to stimulate an intracellular killing response. To examine this possibility, plasmids encoding YopE variants that were defective for membrane localization (YopE3N) [17] or less stable (YopER62K) [19] were introduced into IP6. The resulting strains (Table 1) were used to infect macrophages and detergent extraction assays were used to compare the amounts of YopE, YopE3N and YopER62K that were translocated. The *yopB* mutant IP40, which is defective for Yop translocation, was used to infect macrophages as a negative control. The amount of YopE3N in the soluble fraction was comparable to wild-type YopE, indicating equal translocation of these proteins (Figure 5A, compare lanes 3 and 1). Some YopE proteins with reduced Rho GAP activity are translocated at higher levels as compared to the wild-type protein into epithelial cells infected with *Y. pseudotuberculosis* [31]. We did not observe hypertranslocation of YopE proteins with reduced Rho GAP activity in our experiments, possibly because YopE has a reduced ability to negatively regulate its own translocation into macrophages as compared to epithelial cells. The amount of YopER62K in the soluble fraction was lower compared to wild-type YopE, probably due to decreased stability as a result of increased ubiquitination (Figure 5A, compare lanes 5 and 1). The appearance of a slower migrating band for YopER62K was consistent with ubiquitination (Figure 5A, lane 5). IP6+YopE3N and IP6+YopER62K displayed improved survival in macrophages in comparison to IP6+YopE at 24 h post infection, as determined using immunofluorescence microscopy to detect intracellular *Yersinia* (Figure 5B). The results, quantified by the percentage of macrophages containing fluorescent intracellular *Yersinia* (Figure 5C), or CFU assay (Figure S8A), showed that IP6+YopE3N and IP6+YopER62K had increased bacterial survival compared to IP6+YopE at 24 h post infection. Since membrane localization and stability are important for YopE to efficiently inactivate Rho GTPases, these results provide additional evidence that macrophages sense the inactivation of one or more Rho GTPases, which results in killing of intracellular Yesinia.

**Figure 6:**
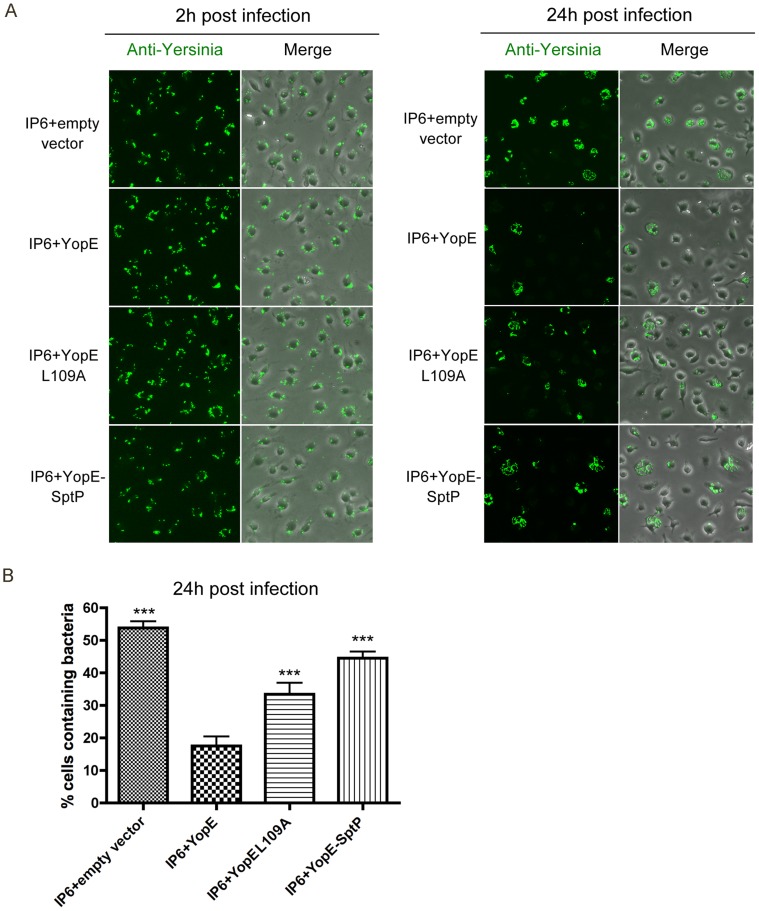
Survival of different *Y. pseudotuberculosis* strains in macrophages as determined by fluorescence microscopy. (A) BMDMs were infected with the indicated strains at an MOI of 10 as described in Figure 1. At 2 h and 24 h post infection, infected cells were fixed, permeabilized and labeled with a rabbit anti-*Yersinia* antibody (green). Shown is bacteria or an overlay of bacteria and phase contrast signal from representative images. (B) Percentage of macrophages containing fluorescent *Yersinia* at 24 h post infection, quantified from three independent microscopy experiments as described in (A). Error bars show standard deviations. ***, P<0.001 compared to IP6+YopE, determined by one-way ANOVA.

YopE variants with altered Rho GTPase specificities [31] were compared to wild-type YopE for their capability to trigger intracellular killing response. YopEL109A has lower GAP activity towards RhoA (70% of wild-type level) and Rac2 (70% of wild-type level); YopE-SptP fusion protein, which contains the secretion and translocation domains of YopE and the GAP domain of SptP, has no GAP activity towards RhoA and decreased activity towards Rac1 (83% of wild-type level) and Rac2 (34% of wild-type level) [31]. The amounts of translocated YopEL109A and YopE-SptP were comparable to wild-type YopE in *Yersinia*-infected macrophages, and YopE-SptP displayed reduced mobility as expected due to its higher molecular weight (Figure 5A, compare lanes 1, 2 and 4). At 24 h post infection, IP6+YopEL109A and IP6+YopE-SptP showed improved survival inside macrophages compared to IP6+YopE, as demonstrated by immunofluorescence microscopy (Figure 6AB) and CFU assays (Figure S8B). These results indicate that the specificity of YopE GAP activity may impact its ability to trigger the intracellular killing. However, the results obtained with the YopEL109A and YopE-SptP variants did not reveal if inactivation of a specific Rho GTPase by YopE is important for intracellular killing. Since the activity of YopEL109A and YopE-SptP towards Rho GTPases other than RhoA, Rac1 and Rac2 is not clearly known, it is difficult to declare YopE interruption of which specific Rho GTPase is essential for macrophage recognition and intracellular killing.

### Toxin B decreases *Yersinia* survival inside macrophages

**Figure 7:**
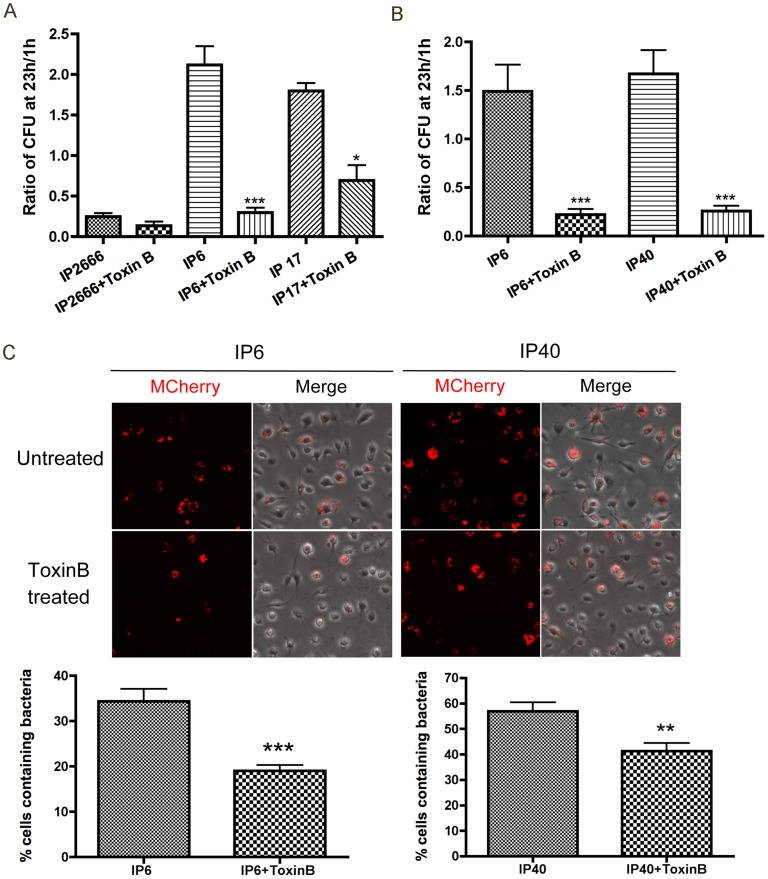
Survival of different *Y. pseudotuberculosis* strains inside macrophages, in the presence or absence of Toxin B. BMDMs were infected with the indicated strains at an MOI of 10 as described in Figure 1. When indicated, 40 ng/ml Toxin B was present throughout the experiment. (A–B) Intracellular bacterial survival was determined by CFU assay, as described in Figure 1. Results shown are the means from four independent experiments with duplicate infection wells. Error bars show standard deviations. ***, P<0.001 and *, P<0.05 comparing each strain with to without Toxin B treatment individually, as determined by one-way ANOVA. (C) Intracellular survival of the indicated mCherry encoding strains was determined by fluorescence microscopy as described in Figure 2. Two hours before fixation, IPTG was added to induce de novo expression of mCherry. The upper panel shows mCherry or an overlay of mCherry and phase contrast signal at 24 h post infection, from representative images. The lower panels show percentage of mCherry positive macrophages quantified from three independent experiments. Error bars show standard deviations. **, P<0.01 and ***, P<0.001 determined by t-test.

To explore if this intracellular killing response applies to bacterial toxins targeting Rho GTPases, *Clostridium difficile* Toxin B was added to *Yersinia* infected macrophages. Toxin B has been well characterized to inactivate a wide range of Rho GTPases through glycosylation, including Rac1, RhoA/B/C, RhoG, TC10, and Cdc42 [32]–[34]. With Toxin B treatment, the survival of IP6, IP17 and IP40 was dramatically decreased as revealed by CFU assays (Figure 7A and B) and fluorescence microscopy in conjunction with mCherry induction (Figure 7C). Toxin B did not affect *Yersinia* growth in tissue culture media in the absence of macrophages; Toxin B did not cause significant cytotoxicity in macrophages in these experiments (data not shown). These results suggest that down-regulation of Rho GTPases by Toxin B is perceived by macrophages, inducing an intracellular killing response, mimicking the effect of YopE. Thus, the bactericidal effect triggered by Rho GTPase-inactivating toxins may be a general and conserved response to these bacterial toxins. In addition, the fact that Toxin B decreases IP40 survival inside macrophages implies that T3SS translocon is not essential for macrophage recognition of Rho GTPases-inactivating toxins to cause a bactericidal response.

### Rac inhibition, but not Rho inhibition, decreases *Yersinia* survival inside macrophages

**Figure 8:**
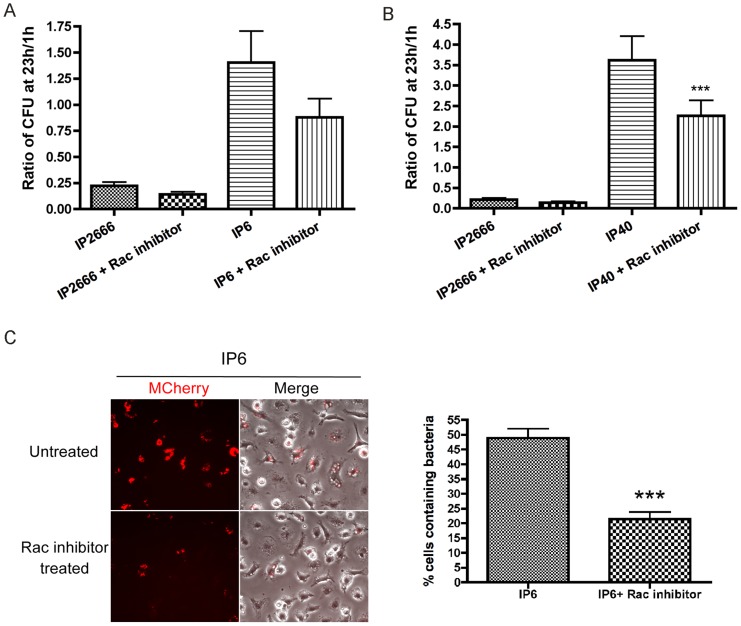
Survival of different *Y. pseudotuberculosis* strains inside macrophages, in the presence or absence of Rac inhibitor NSC23766. BMDMs were infected with the indicated strains at an MOI of 10 as described in Figure 1. When indicated, 100 µM NSC23766 was present throughout the experiment. (A–B) Intracellular bacterial survival was determined by CFU assay, as described in Figure 1. Results shown are the means from three independent experiments with duplicate infection wells. Error bars show standard deviations. ***, P<0.001, comparing each strain with to without Rac inhibitor treatment individually, as determined by one-way ANOVA. (C) Intracellular survival of mCherry-encoding IP6 was determined by fluorescence microscopy, as described in Figure 7C. The left panel shows mCherry or an overlay of mCherry and phase contrast signal at 24 h post infection from representative images. The right panel shows percentage of mCherry positive macrophages quantified from three independent experiments. Error bars show standard deviations. ***, P<0.001 determined by t-test.

To identify the Rho GTPase target of YopE critical for causing intracellular killing, specific Rho GTPase inhibitors were studied for their capability to mimic the YopE effect. Treatment with Rac1 inhibitor NSC23766 negatively impacted IP6 and IP40 survival inside macrophages, as demonstrated by CFU assays (Figure 8AB) and fluorescence microscopy with mCherry induction (Figure 8C). The Rac1 inhibitor triggered a reduced bactericidal effect in comparison to Toxin B in the CFU assay (compare Figure 7AB and 8AB). In contrast to the Rac1 inhibitor, the RhoA inhibitor TAT-C3 did not significantly affect *Yersinia* survival inside macrophages (Figure S9BC). Dramatic morphological changes were observed in TAT-C3 treated macrophages as early as 4 h upon treatment, confirming the efficiency of TAT-C3 towards RhoA (Figure S9A). These results signify that macrophages restrict *Yersinia* intracellular survival in response to Rac1 inhibition, but not to Rho inhibition.

### Presence of YopE induces higher levels of nitric oxide from infected macrophages

Several recent studies have shown that the activities of certain bacterial effectors can stimulate transcriptional changes in host cells, resulting in ETIRs [5]–[9]. For example, activation of Rac1 and Cdc42 by SopE from *Salmonella enterica* serovar Typhimurium is sensed through NOD1 receptor, eliciting NF-κB activation in the host cells as a protective response [9].

**Figure 9:**
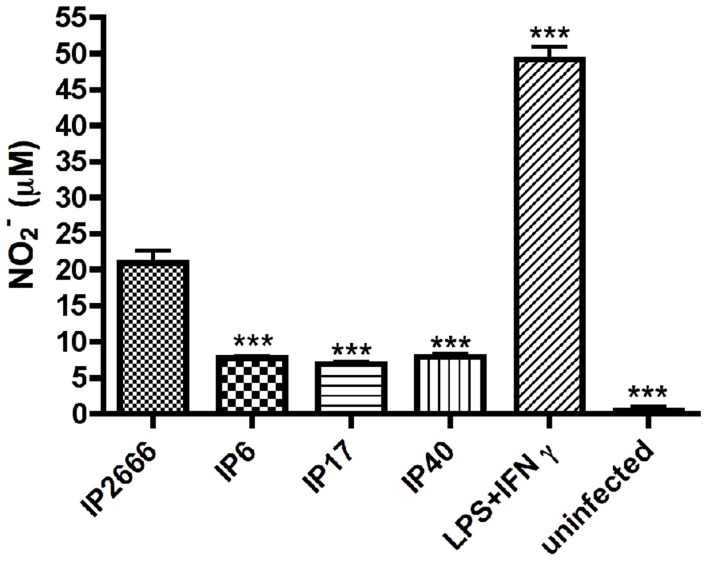
Measurement of NO production by macrophages infected with different *Y. pseudotuberculosis* strains. BMDMs were infected with the indicated strains at an MOI of 10 as described in Figure 1. At 23 h post infection, medium from the infected macrophages were collected and subjected to Griess assay to determine nitrite (NO_2^−^_) concentrations. Lipopolysaccharide (LPS)- and IFN γ- treated macrophages are shown as a positive control. Results shown are the means from three independent experiments with duplicate infection wells. Error bars show standard deviations. ***, P<0.001, comparing to IP2666 infected macrophages, as determined by one-way ANOVA.

To study if YopE stimulates an altered host response that can occur at the transcriptional level, the production of nitric oxide (NO) from macrophages infected with IP2666, IP6, IP17 or IP40 was compared. Specifically, the concentration of nitrite (NO_2^−^_), an indicator of NO, was measured by Griess assay. At 23 h post infection, comparing to IP6-, IP17- or IP40-infected macrophages, IP2666-infected macrophages produced significantly higher levels of NO (Figure 9). LPS- and IFN γ-treated macrophages were used as a positive control, while uninfected macrophages were used as a negative control (Figure 9).

To investigate whether YopE dependent-intracellular killing signals through NOD1 receptor, *Nod1^−/−^* BMDMs were compared to wild-type BMDMs for their ability to restrict intracellular survival of IP2666 or IP6 by CFU assay. No significant difference was observed for IP2666 survival inside wild-type or *Nod1^−/−^* BMDMs (Figure S10).

These results suggest that macrophages respond to wild-type *Yersinia* differently from *yopE^−^* mutant strains and produce higher levels of NO; however, YopE-triggered intracellular killing is not mediated by NOD1 receptor.

## Discussion

**Figure 10:**
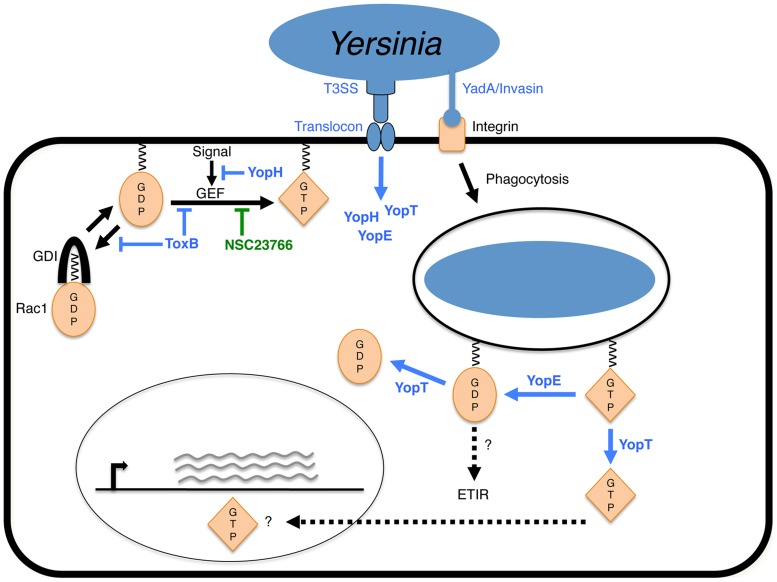
Model of YopE-triggered killing of *Yersinia* in macrophages. Binding of *Y. pseudotuberculosis* to macrophage β1 integrin via YadA or Invasin allows for translocation of Yop effectors by the type III secretion system (T3SS) and initiates phagocytosis. Regulation of Rac1 activation by host guanine nucleotide dissociation inhibitor (GDI) or guanine nucleotide exchange factor (GEF) is shown at the plasma membrane. For simplicity, inhibition of Rac1 activation by YopH, Toxin B or NSC23766 is shown at the plasma membrane and manipulation of Rac1 by YopE and YopT is shown on phagosomal membrane. Production of membrane-localized inactive Rac1-GDP is proposed to generate an effector-triggered immune response (ETIR), resulting in increased killing of *Yersinia* in phagosomes. YopT counteracts the ETIR by releasing GDP-bound Rac1 from the membrane. Additionally, activated GTP-bound Rac1 released from the membrane by YopT has been shown to localize to the nucleus and may activate gene expression.

The aims of this study were to determine T3SS-dependent factors that restrict *Yersinia* survival inside macrophages and characterize the mechanism of this “patterns of pathogenesis” triggered host response. Our findings reveal that primary naïve macrophages sense manipulation of Rho GTPases by *Yersinia* Yop effectors. Three known effector Yops directly inhibit host Rho GTPases: YopE, YpkA and YopT; a fourth effector, YopH, inhibits signals that activate these small GTPases. YopE is an important virulence factor for resistance of *Yersinia* to the innate immunity, as a *Y. pseudotuberculosis yopE* null mutant was defective for systemic spread following oral infection in the animal model [35]. However, on the other hand, here we show that YopE GAP activity towards Rho GTPases is recognized by macrophages, stimulating increased killing of intracellular *Y. pseudotuberculosis* (Figure 10). YopH cooperates with YopE to cause this killing effect, most likely by inhibiting a phosphotyrosine dependent signaling pathway that activates Rho GTPases in response to *Yersinia* infection (Figure 10). YpkA has very mild effect on *Yersinia* intracellular survival (data not shown), perhaps due to its low expression level in comparison to other Yops. Interestingly, we have observed that overexpression of YopT counteracts the YopE-triggered intracellular killing effect, which involves the protease activity of YopT. We speculate that an important biological function of YopT is to counteract sensing of YopE by the innate immune system, possibly by preventing YopE access to activated Rho GTPase targets or removing YopE-inactivated Rho GTPases from phagosome membranes (Figure 10). Zhang *et al.* studied a *Y. pseudotuberculosis* strain (32777), different from that used here (IP2666), and showed that a mutant of 32777 encoding catalytically inactive YopJ, YopT, YopE and YopH still triggered intracellular bacterial killing, to the same level as wild-type 32777 [25]. We speculate that 32777 has additional Rho GTPase-inactivating effector(s) causing bacterial killing, which remain to be identified.

We have obtained evidence that Toxin B decreases *Yersinia* survival in macrophages by inactivating several Rho GTPases (Figure 10). To date, at least 20 Rho GTPase proteins belonging to 8 subfamilies have been described in mammals [36], [37]. Interestingly, we have shown that the target preference of YopE impacts its ability to trigger bacterial killing. Thus, one intriguing question to ask is does the innate immune system monitor effector manipulation of a specific Rho GTPase? Our results showed that Rac inhibition, but not Rho inhibition, stimulates the macrophage killing response against intracellular *Yersinia* (Figure 10). However, the Rac inhibitor only partially promotes killing of *Yersinia* in macrophages in comparison to Toxin B or YopE, suggesting that disturbance of additional Rho GTPases contributes to the intracellular killing response. Other Rho GTPase candidates may include, but are not limited to, Rac2 and RhoG, which have been shown to be YopE targets and expressed in macrophages [31], [37]–[39]. Further investigation is required to reveal if additional Rho GTPases serve as surveillance points in response to pathogenic effector manipulation [37].

Rho GTPases act as molecular switches that regulate numerous cellular functions, like cytoskeletal dynamics, gene transcription, vesicular trafficking, cell growth and apoptosis [37], [40]. In order to ensure proper signaling responses, the activities of Rho GTPases are tightly regulated by multiple mechanisms, including the canonical regulators (GAPs, GEFs and GDIs) and direct post-translational modifications (like phosphorylation and ubiquitination) [40], [41]. The abnormal inactivation of Rho GTPases by YopE might interfere with multiple Rho GTPase-mediated signaling pathways and lead to many different consequences to trigger intracellular bacteria killing in macrophages. One possibility is that YopE may cause accumulation of inactivated GDP-bound Rho GTPases on phagosome (Figure 10), which could be modified by ubiquitination to stimulate signaling pathways. Activation of Rac1 by cytotoxic necrotizing factor 1 (CNF1) from *Escherichia coli* induces Rac1 poly- and mono-ubiquitination, the biological function of the latter remains unclear [42]. In line with this, GDP-bound RhoA is targeted by the ubiquitin E3 ligase Cullin-3 for poly-ubiquitination and degradation [43]. Thus, it is tempting to speculate that YopE-inactivated GDP-bound Rho GTPases could be mono-ubiquitinated and serve as signaling components; or they could be poly-ubiquitinated to mediate xenophagic degradation of bacteria-containing vesicles [44]. Alternatively, by modulating vesicular trafficking, YopE activity may interrupt formation of *Y. pseudotuberculosis*-containing autophagosomes, which have been shown to be impaired in acidification and support survival of the bacteria in macrophages [45]. On the other hand, given that the role of autophagy in *Yersinia* survival in macrophages is controversial [45], [46], it is possible that YopE activity promotes autophagy to eliminate intracellular bacteria. Deuretzbaher et *al.* showed that β1-integrin-mediated *Y. enterocolitica* internalization by macrophages was coupled to autophagy activation, which seemed to be deleterious for bacterial intracellular survival. Another possibility is that the disruption of the actin cytoskeleton by YopE is sensed by the innate immune system. It has been suggested that NOD receptors or inflammasome components associated with the actin cytoskeleton may act as surveillance mechanisms, becoming activated upon perturbations by pathogens [2]. Interesting, a recent study by Shao and colleagues showed that Rho-inactivating toxins such as *Clostridium difficile* Toxin B and *Clostridium botulinum* C3 trigger Pyrin inflammasome activation in BMDMs [47]. They further demonstrated that *Burkholderia cenocepacia* induced inactivation of Rho GTPase stimulates Pyrin inflammasome activation as an immune defense, which limits bacterial intra-macrophage growth and regulates lung inflammation in infected mice [47]. Whether YopE triggers *Yersinia* intracellular killing through the inflammasome pathway remains to be investigated.

The overall host cell innate immune response to a T3SS-containing bacterial pathogen is unique and multifactorial. MAMPs, the T3SS translocon channel, and the activities of bacterial effectors are likely recognized as combined pathogenic signals by the host cell. A two-signal model, requiring a MAMP and a pattern of pathogenesis, was proposed as an innate immune strategy to evaluate the virulence potential of a pathogen and adjust immune response appropriately to avoid self-damaging inflammation [48]. For example, a type IV secretion system allows *Legionella pneumophila* to deliver bacterial effectors into the host cell cytosol to inhibit host protein synthesis [6]. In this case, the effector-mediated interference of host protein synthesis, in concert with TLR signaling, results in prolonged activation of NF-κB as an ETIR [6]. Our data suggest that the YopBD translocon is not essential for Toxin B- or Rac inhibitor-triggered bacterial killing in macrophages. Further studies are needed to determine if TLRs or β1-integrins are possible receptors in MAMP-PRR pathways that facilitate the YopE-triggered killing effect. Alternatively, some studies in the literature support the idea that patterns of pathogenesis are sufficient to induce defense responses independently of classical MAMPs [5], [49], [50]. Boyer et *al.* demonstrated that *Escherichia coli* CNF1 elicited a vigorous ETIR in flies via activation of Rac2 and the IMD kinase pathway, which was observed even in the absence of PRR ligation [5]. Thus, it is possible that unbalanced disruption of Rho GTPases by YopE is adequate to stimulate a protective immune response, resulting in restriction of *Yersinia* survival in macrophages.

Roy and colleagues observed that during internalization of *Salmonella enterica* serovar Typhimurium or *Y. pseudotuberculosis*, the T3SSs of these pathogens stimulated SytVII-dependent phagolysosome fusion and bacterial killing in macrophages [26]. We have shown that YopE-triggered intracellular bacterial killing does not require SytVII, suggesting that there are at least two independent pathways by which killing of *Yersinia* internalized by macrophages can be stimulated. The YopE-dependent pathway senses inactivation of Rho GTPases, while the SytVII dependent pathway appears to recognize translocon insertion in the plasma membrane.

Various bacterial T3SS and T4SS effectors modulate Rho GTPase functions and interfere with corresponding host signaling pathways to benefit pathogenic infection [51]. Given that Rho GTPases play multiple roles in many signaling pathways critical for cellular functions, it is not surprising to envision surveillance mechanisms monitoring the status of Rho GTPases. Our work highlights that inactivation of Rac1, and possibly other GTPases, by YopE from *Y. pseudotuberculosis* is detected by macrophages as a danger signal, stimulating an ETIR that restricts intracellular bacterial survival. Detection of pathogens via Rho GTPase surveillance adds another layer of complexity to the mechanisms of innate immune recognition, improving our understanding of how the innate immune system responds to pathogenic infection.

## Materials and Methods

### Ethics statement

Use of mice for the preparation of BMDMs was carried out in accordance with a protocol that adhered to the Guide for the Care and Use of Laboratory Animals of the National Institutes of Health (NIH) and was reviewed and approved (approval #206152) by the Institutional Animal Care and Use Committee at Stony Brook University, which operates under Assurance #A3011-01, approved by the NIH Office of Laboratory Animal Welfare.

### Bacterial strains and plasmid constructions

The *Y. pseudotuberculosis* strains used in this study are shown in Table 1. These bacteria were grown on LB agar plates or in LB broth at 28°C supplemented with 100 µg/ml ampicillin, 25 µg/ml kanamycin or 30 µg/ml chloramphenicol as needed. The plasmids pMMB67HE [52], pYopE [14], pYopER144A [14], pPEYopE [30], pYopT [53], pPTYopT [30], pYopTC139S [18], p67GFP3.1 [23] and p207mCherry [54] have been previously described.

A new series of plasmids expressing *yopE* mutants were created as described below. Plasmids encoding *yopEL109A*, *yopER62K* and *yopE3N* were generated as follows. DNA fragments encoding *yopEL109A*, *yopER62K* or *yopE3N* were obtained by PCR using primers yopE-3 (5′-CGGATCCCATATGAAAATATCATCATTTATTTC-3′) and yopE-EcoRI (5′-CGCGGAATTCCCATATCACATCAATGACAGTAATTT-3′). Recombinant plasmid DNA (pBAD33/YopEL109A, a gift from Joan Mecsas, Tufts University), or *Y. pseudotuberculosis* virulence plasmid DNA (from 32777 *yopER62K* or 32777 *yopE3N*, Zhang et al. submitted) was used as template for the PCR to obtain *yopEL109A*, *yopER62K* and *yopE3N*, respectively. The resulting DNA fragments were inserted into pETBlue2 vector using blunt end ligation. To create a plasmid encoding the *yopE-sptP fusion*, a DNA fragment containing the first 100 codons of *yopE* (*yopE_1–100_*) was amplified from IP2666 virulence plasmid DNA with primers yopE-infusion-5 (5′-TAATAAATAGTCATATGAAAATATCATCATTTATTTCTACATCACTG-3′) and yopE-infusion-3 (5′-AGGTTGCTTACTTTCCGTAGGACTTGGCATTTGTGC-3′). A DNA fragment containing codons 166–293 of *sptP* (*sptP_166–293_*) was amplified with primers sptP-infusion-5 (5′-ATGCCAAGTCCTACGGAAAGTAAGCAACCTTTACTCAGTATCG-3′) and sptP-infusion-3 (5′-CAGCCAAGCTGAATTTTAGCCGGCTTCTATTTTCTCAAGTTC-3′) using chromosomal DNA from *Salmonella enterica* Typhimurium strain 14028 as template. A DNA fragment encoding the *yopE_1–100_sptP_166–263_* fusion was made by overlapping PCR using the *yopE_1–100_* and *sptP_166–293_* fragments as templates and primers yopE-infusion-5 and sptP-infusion-3. The product was inserted into pETBlue2 by blunt end ligation. The sequences of the inserts in the plasmids described above were confirmed by DNA sequencing. DNA fragments encoding *yopEL109A*, *yopER62K*, *yopE3N* or *yopE_1–100_sptP_166–263_* were obtained from the pETBlue2 vectors by digestion with NdeI and EcoRI, and ligated between the NdeI and EcoRI sites in pPEYopE, thereby replacing the wild-type *yopE* gene, and placing the mutant alleles under control of the native *yopE* promoter. The resulting plasmids pYopEL109A, pYopER62K, pYopE3N and pYopE-SptP were introduced into *E. coli* S17-1 λpir by electroporation and subsequently transferred into IP6 (Table 1) by conjugation as described previously [55].

### Cell culture

Bone marrow-derived macrophages (BMDMs) were isolated and cultured from femurs of C57BL/6 wild-type mice (Jackson Laboratory) or *SytVII^−/−^* C57BL/6 mice (a generous gift from Dr. Norma Andrews, University of Maryland), or *Nod1^−/−^* C57BL/6 mice (a generous gift from Dr. Andreas Baumler, University of California-Davis) as previously described [56]. 24 h before infection, macrophages were seeded into 24-well tissue culture plate at a density of 1.5×10^5^ cells/well in Dulbecco's modified Eagle medium supplemented with 10% fetal bovine serum (Hyclone), 15% L-cell conditioned medium, 1 mM sodium pyruvate and 2 mM glutamate.

### Infection conditions

*Y. pseudotuberculosis* strains were grown at 28°C in LB broth with aeration overnight. The next day, overnight cultures were diluted 1∶40 into fresh LB broth containing 2.5 mM CaCl_2_ and sub-cultured at 37°C for 2 h to induce *yop* gene expression. Bacteria were washed once and resuspended in HBSS to obtain optical density at OD 600 nm. Next, bacteria were diluted into cell culture medium to infect macrophages at an MOI of 10, unless specified. After centrifugation for 5 min at 700 rpm to facilitate bacterial contact with macrophages, another 15 min incubation was performed at 37°C, giving the total infection time of 20 min. The end of 20 min incubation is considered as 0 h post infection. To eliminate extracellular bacteria, unless specified, the cells were then incubated in medium containing 8 µg/ml gentamicin for 1 h, and then maintained in fresh medium containing 4.5 µg/ml gentamicin until the end of incubation. When indicated, 40 ng/ml Toxin B (Calbiochem), 100 µM NSC23766 (Calbiochem), or 10 µg/ml TAT-C3 was added at 0 h post infection and maintained throughout the experiment. TAT-C3 was purified and kindly provided by Dr. Gloria Viboud, Stony Brook University [57].

### CFU assay

At the time points indicated in the figures, the infected BMDMs were washed twice with HBSS, lysed and scraped with 500 µl 0.1% Triton X-100 in HBSS to release intracellular bacteria. After collecting the lysates, 500 µl HBSS was used to rinse the wells and collect any residual bacteria. The lysates and the wash were combined, serially diluted and spread on LB plates, and then incubated at 28°C for 2 days to enumerate output CFU.

### Immunoblotting

The primary antibodies used were a cocktail of two monoclonal mouse anti-YopE antibodies designated 202 and 149 (unpublished data), the monoclonal mouse anti-YopH antibody designated 3D10 (a gift from Dr. Richard Siegel, NIH) diluted 1∶1000, a polyclonal rabbit anti-YopT antibody diluted 1∶500 [30], and a polyclonal rabbit anti-β-actin antibody (Cell signaling) diluted in 1∶1000. The secondary antibodies used were a goat anti-mouse antibody conjugated to IRD800 (Rockland) diluted 1∶5000 and a donkey anti-rabbit antibody conjugated to IRD800 (Rockland) diluted 1∶5000.

The protein samples were separated by sodium dodecyl sulfate-polyacrylamide gel electrophoresis, transferred to nitrocellulose membranes, and subjected to immunoblotting with specific primary and secondary antibodies. The membranes were then scanned and analyzed with the Odyssey system (Li-Cor Biosciences).

### Fluorescence microscopy

BMDMs were prepared and infected as described above, except that they were seeded into wells with glass coverslips, which had been washed with acetone and heated at 180°C for 4 h to remove LPS. At indicated time points, infected BMDMs were washed three times with PBS and fixed with 2.5% PFA for 10 min. When needed, 0.5 mM Isopropyl-β-D-thiogalactopyranoside (IPTG) was added at 1 h before fixation to induce de novo GFP expression, or at 2 h before fixation to induce de novo mCherry expression. When indicated, immunofluorescence staining was performed as described previously [23]. Briefly, the fixed cells were permeabilized with 0.1% Triton X-100 in PBS for 1 min, followed by blocking with 3% bovine serum albumin in PBS for 10 min. The cells were then incubated with a polyclonal rabbit anti-Yersinia antibody SB349 diluted 1∶1000 for 30 min. After washing with PBS, the cells were incubated with FITC conjugated anti-rabbit antibody (Jackson Laboratories) diluted 1∶250 for 40 min. After washing, the coverslips were inverted onto 6 µl Prolong Gold anti-fade reagent (Invitrogen) on a microscope slide. The slides were examined by fluorescence microscopy using a Zeiss Axioplan2 microscope with a 32× objective. Three randomly selected fields of each slide were examined. In each field, about 50 BMDMs were examined from merged images of phase contrast and fluorescence, which were captured with a Spot camera (Diagnotic Instruments, Inc) and processed with Adobe Photoshop. Percentage of cells containing bacteria was quantified using three independent experiments.

### Detergent extraction assay

Detergent extraction assays were performed as previously described in [58]. BMDMs were infected as described above, except that they were seeded in 6 well plates at a density of 8×10^5^ cells/well and infected at an MOI of 30 for 2 h. The infected cells were washed twice with ice-cold HBSS and lysed with 50 µl 1% Triton X-100 in HBSS containing EDTA-free protease inhibitor cocktail (ROCHE). After 10 min on ice, the cells were scraped from the plate to collect the lysates. The soluble and insoluble fractions of the lysates were separated by centrifugation (14000 rpm, 10 min, 4°C) and subsequently analyzed using immunoblotting as described above.

### Tail genotyping

Chromosome DNA was isolated from C57BL/6 wild-type or *SytVII^−/−^* mouse tails and used as templates for PCR amplification. Briefly, tail tips were digested in 500 µl lysis buffer (0.1M NaCl, 0.05M Tris-HCL pH 7.7, 1% SDS and 2.5 mM EDTA) with 40 µg/ml freshly added proteinase K (Sigma), and incubated at 55°C overnight. The resulting supernatant were collected and mixed with 500 µl isopropanol to precipitate chromosomal DNA. After centrifugation (14000 rpm, 10 min, RT), the pellets were washed twice with 70% ethanol, air-dried for 5 min, and dissolved in 100 µl TE buffer. Genotyping PCR were performed with the following primers: P1 (5′-CATCCTCCACTGGCCATGAATG-3′), P2 (5′-GCTTCACCTTGGTCTCCAG-3′), P3 (5′-CTTGGGTGGAGAGGCTATTC-3′) and P4 (5′-AGGTGAGATGACAGGAGATC-3′). PCR products were analyzed by agarose gel electrophoresis.

### GRIESS assay

NO levels generated by infected macrophages were determined by measuring the accumulation of nitrite (NO_2^−^_) using the Griess assay as described previously [59]. Control macrophages were treated with *E.coli* LPS (100 µg/µl, Sigma) and IFN γ (0.1 units/µl, ROCHE) throughout the experiment. At 23 h post infection, conditioned medium were collected and centrifuged (14000 rpm, 10 min, RT). 100 µl of the supernatant was mixed with 100 µl Griess reagent (0.5% sulfanilamide and 0.05% N-(1-naphthyl)ethylenediamide in 2.5% acetic acid) and incubated for 10 min at room temperature. The samples were then measured at OD_550 nm_. The concentration of NO_2^−^_ was calculated by using a standard curve prepared with sodium nitrite.

### Accession numbers

The GenBank accession number for the YopE protein studied in this work is CAA68609.1.

## Supporting Information

Figure S1 Survival of *Y. pseudotuberculosis* inside macrophages determined by CFU assay, as described in Figure 1. (A) The logarithm of intracellular bacteria count per well at 1 h post infection. (B) The logarithm of intracellular bacteria count per well at 23 h post infection. Results shown are the means from four independent experiments with duplicate infection wells. Error bars show standard deviations. *, P<0.05 and ***, P<0.001 compared to IP17, as determined by one-way ANOVA.
(TIF)

Figure S2 Survival of *Y. pseudotuberculosis* inside macrophages determined by CFU assay. BMDMs were infected with the indicated strains as described in Figure 1, except that indicated MOIs were used. (A) The logarithm of intracellular bacteria count per well at 1 h and 23 h post infection. (B) Ratio of CFU at 23 h/1 h. Results shown are the means from three independent experiments with duplicate infection wells. Error bars show standard deviations. *, P<0.05 and ***, P<0.001 compared to IP2666 at an MOI of 10, as determined by one-way ANOVA.
(TIF)

Figure S3 Survival of different *Y. pseudotuberculosis* strains in wild-type or *SytVII^−/−^* macrophages. (A) Verification of *SytVII^−/−^* mice by tail genotyping. Shown are PCR results obtained with mouse-tail genomic DNA using indicated primers. Wild type = 400 bp; mutant = 280 bp. (B) Wild-type or *SytVII^−/−^* BMDMs were infected with the indicated strains. Intracellular bacterial survival was measured by CFU assay, as described in Figure 1. Results shown are the means from three independent experiments with duplicate infection wells. Error bars show standard deviations. There is no significant difference in the survival of each strain in WT BMDMs as compared individually to that in *SytVII^−/−^* BMDMs.
(TIF)

Figure S4 Survival of *Y. pseudotuberculosis* inside macrophages determined by CFU assay, as described in Figure 1. After 20 min infection, infected cells were incubated in medium with 8 µg/ml, 16 µg/ml or 32 µg/ml gentamicin until the end of the experiment, except the first experimental group, which were incubated in medium with 8 µg/ml gentamicin for 1 h and switched in medium with 4.5 µg/ml gentamicin until the end of the experiment. Shown are the logarithms of intracellular bacteria count per well at 1 h and 23 h post infection. IP2666 is shown in squares; IP17 is shown in triangles. Results shown are the means from three independent experiments with duplicate infection wells. Error bars show standard deviations.
(TIF)

Figure S5 Survival of *Y. pseudotuberculosis* strains with or without empty vector inside macrophages determined by CFU assay, as described in Figure 1. Results shown are the means from three independent experiments with duplicate infection wells. Error bars show standard deviations. *, P<0.05, comparing each strain with to without empty vector individually, as determined by one-way ANOVA.
(TIF)

Figure S6 Survival of *Y. pseudotuberculosis* inside macrophages determined by CFU assay as described in Figure 1. (A) The logarithm of intracellular bacteria count per well at 1 h post infection and 23 h post infection. **, P<0.01 and ***, P<0.001 compared to IP2666+empty vector. (B) Ratio of CFU at 23 h/1 h. **, P<0.01 compared to IP6+empty vector. (C) Ratio of CFU at 23 h/1 h. ***, P<0.001 compared to IP37+empty vector. Results shown are the means from at least three independent experiments with duplicate infection wells. Error bars show standard deviations.
(TIF)

Figure S7 Measurement of YopE translocation in macrophages by different *Y. pseudotuberculosis* strains. (A) YopE translocation levels in BMDMs infected by the indicated strains, determined by detergent extraction assay and immunoblotting analysis using anti-YopE antibodies, as described in Figure 5. β-actin levels from the soluble fraction are shown as loading controls. (B) The intensity of each band was calculated using Odyssey IR imaging system, and the YopE/β actin ratios were normalized according to IP2666+empty vector. Results shown are the means from two independent experiments. Error bars show standard deviations.
(TIF)

Figure S8 Survival of *Y. pseudotuberculosis* strains determined by CFU assay as described in Figure 1. Shown is the logarithm of intracellular bacteria count per well at 1 h post infection and 23 h post infection comparing IP6+empty vector, IP6+YopE, IP6+YopE3N and IP6+YopER62K (A) or IP6+empty vector, IP6+YopE, IP6+YopEL109A and IP6+YopE-SptP (B). Results are the means from three independent experiments with duplicate infection wells. Error bars show standard deviations. *, P<0.05; **, P<0.01 and ***, P<0.001 compared to IP6+YopE, as determined by one-way ANOVA.
(TIF)

Figure S9 Survival of *Y. pseudotuberculosis* strains inside macrophages, in the presence or absence of TAT-C3. (A) Morphological changes of BMDMs upon TAT-C3 treatment was determined by phase contrast microscopy. Shown are BMDMs treated with 10 µg/ml TAT-C3 for 4 h or 24 h. BMDMs with no treatment are also shown as controls. (B) Intracellular bacterial survival was determined by CFU assay, as described in Figure 1. When indicated, 10 µg/ml TAT-C3 was present throughout the experiment. Results shown are the means from three independent experiments with duplicate infection wells. There is no significant difference in the survival of each strain with TAT-C3 treatment as compared individually to that without treatment. (C) Intracellular survival of mCherry encoding IP6 was determined by fluorescence microcopy, as described in Figure 7. When indicated, 10 µg/ml TAT-C3 was added throughout the experiment. Shown is mCherry or an overlay of mCherry and phase contrast signal at 24 h post infection.
(TIF)

Figure S10 Survival of different *Y. pseudotuberculosis* strains in wild-type or *Nod1^−/−^* macrophages. Wild-type or *Nod1^−/−^* BMDMs were infected with the indicated strains at an MOI of 10. Intracellular bacterial survival was measured by CFU assay, as described in Figure 1. (A) The logarithm of intracellular bacteria count per well at 1 h post infection and 23 h post infection. (B) Ratio of CFU at 23 h/1 h. Results shown are the means from three independent experiments with duplicate infection wells. Error bars show standard deviations. There is no significant difference in the survival of each strain in WT BMDMs as compared individually to that in *Nod1^−/−^* BMDMs.
(TIF)
