# Supplementary figures and images for: The GAP Activity of Type III Effector YopE Triggers Killing of Yersinia in Macrophages
Source: PLoS Pathog. 2014 Aug 28;10(8):e1004346. doi: 10.1371/journal.ppat.1004346 (PMC4148447; doi:10.1371/journal.ppat.1004346)

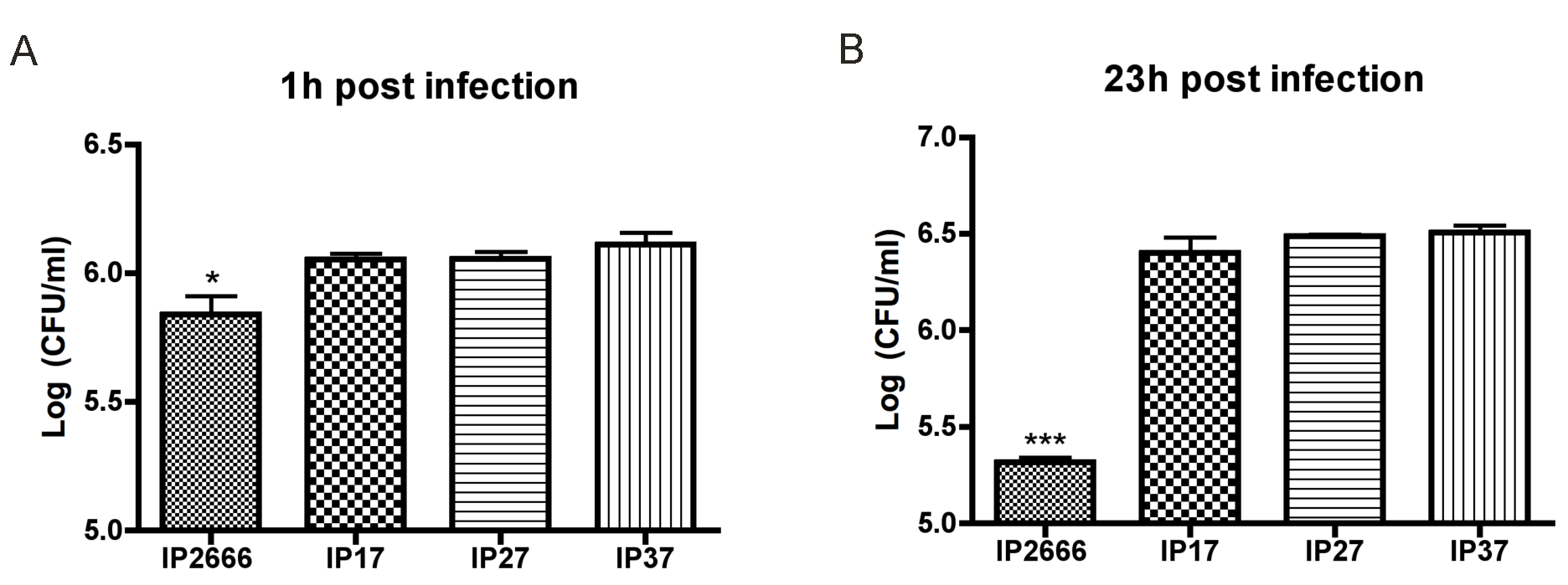

Supplement: Figure S1 — Survival of Y. pseudotuberculosis inside macrophages determined by CFU assay, as described in Figure 1. (A) The logarithm of intracellular bacteria count per well at 1 h post infection. (B) The logarithm of intracellular bacteria count per well at 23 h post infection. Results shown are the means from four independent experiments with duplicate infection wells. Error bars show standard deviations. *, P<0.05 and ***, P<0.001 compared to IP17, as determined by one-way ANOVA. (TIF) [file ppat.1004346.s001.tif]

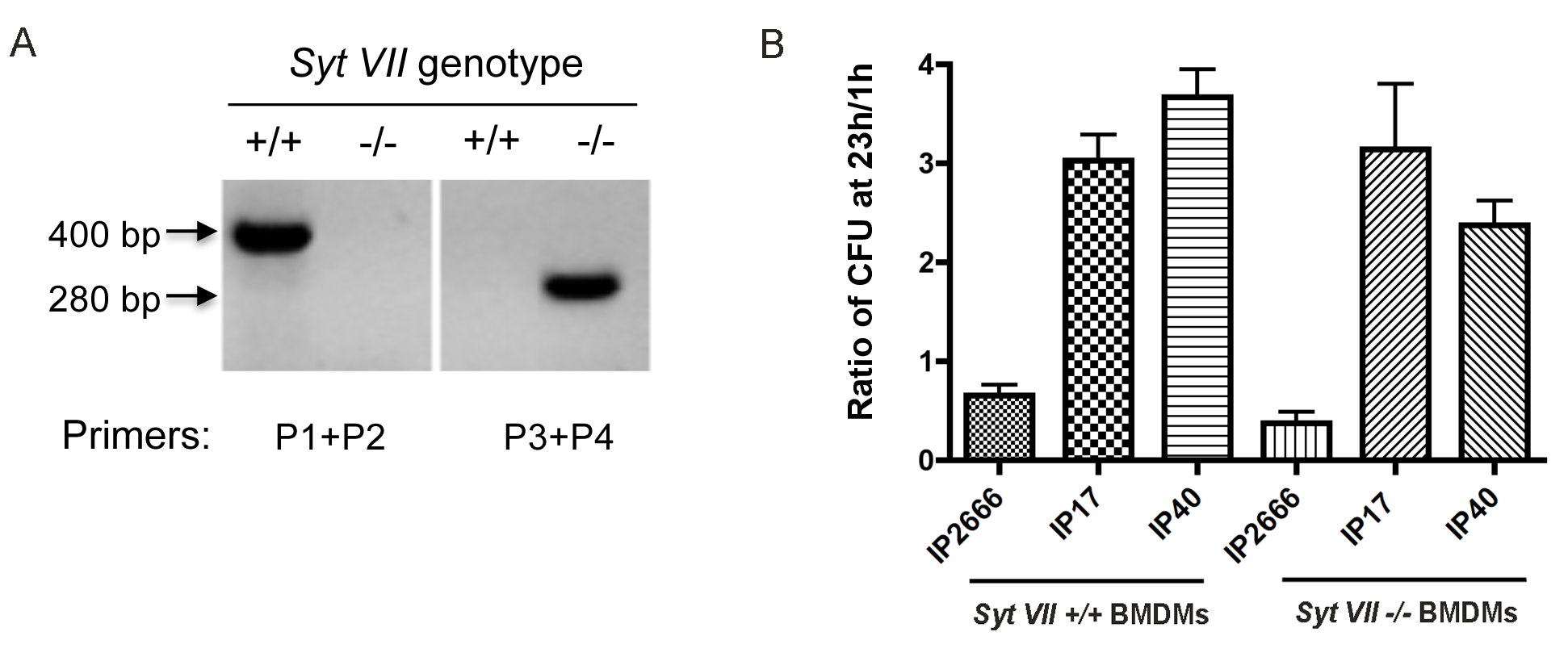

Supplement: Figure S3 — Survival of different Y. pseudotuberculosis strains in wild-type or SytVII−/− macrophages. (A) Verification of SytVII−/− mice by tail genotyping. Shown are PCR results obtained with mouse-tail genomic DNA using indicated primers. Wild type = 400 bp; mutant = 280 bp. (B) Wild-type or SytVII−/− BMDMs were infected with the indicated strains. Intracellular bacterial survival was measured by CFU assay, as described in Figure 1. Results shown are the means from three independent experiments with duplicate infection wells. Error bars show standard deviations. There is no significant difference in the survival of each strain in WT BMDMs as compared individually to that in SytVII−/− BMDMs. (TIF) [file ppat.1004346.s003.tif]

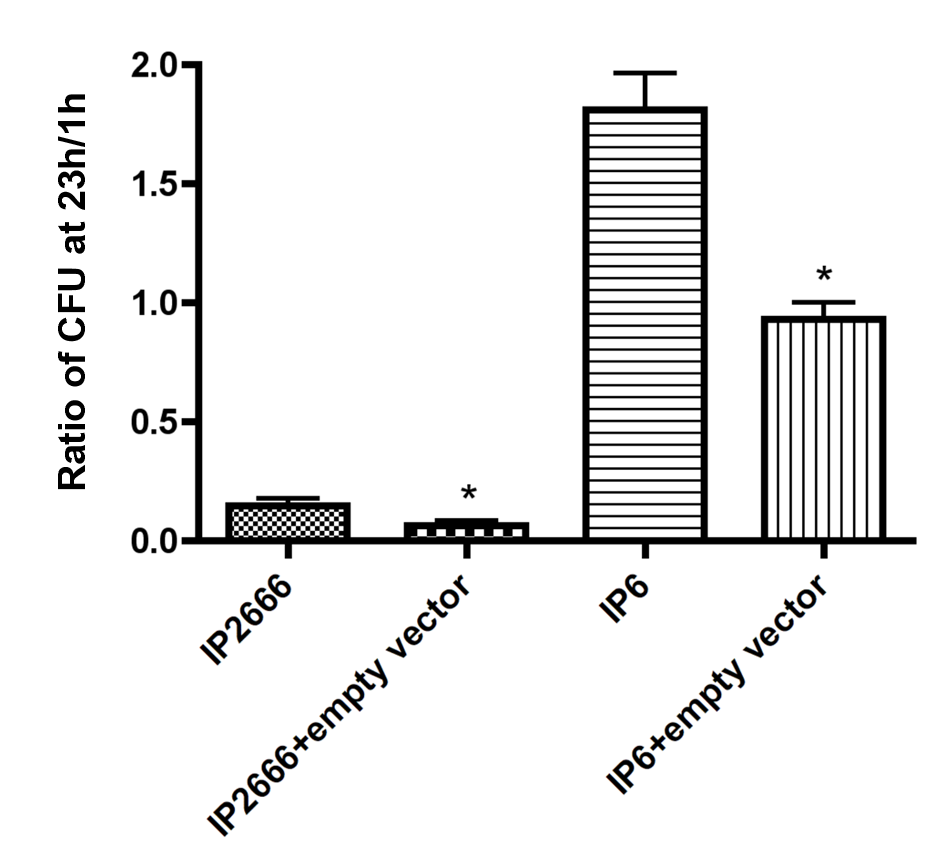

Supplement: Figure S5 — Survival of Y. pseudotuberculosis strains with or without empty vector inside macrophages determined by CFU assay, as described in Figure 1. Results shown are the means from three independent experiments with duplicate infection wells. Error bars show standard deviations. *, P<0.05, comparing each strain with to without empty vector individually, as determined by one-way ANOVA. (TIF) [file ppat.1004346.s005.tif]

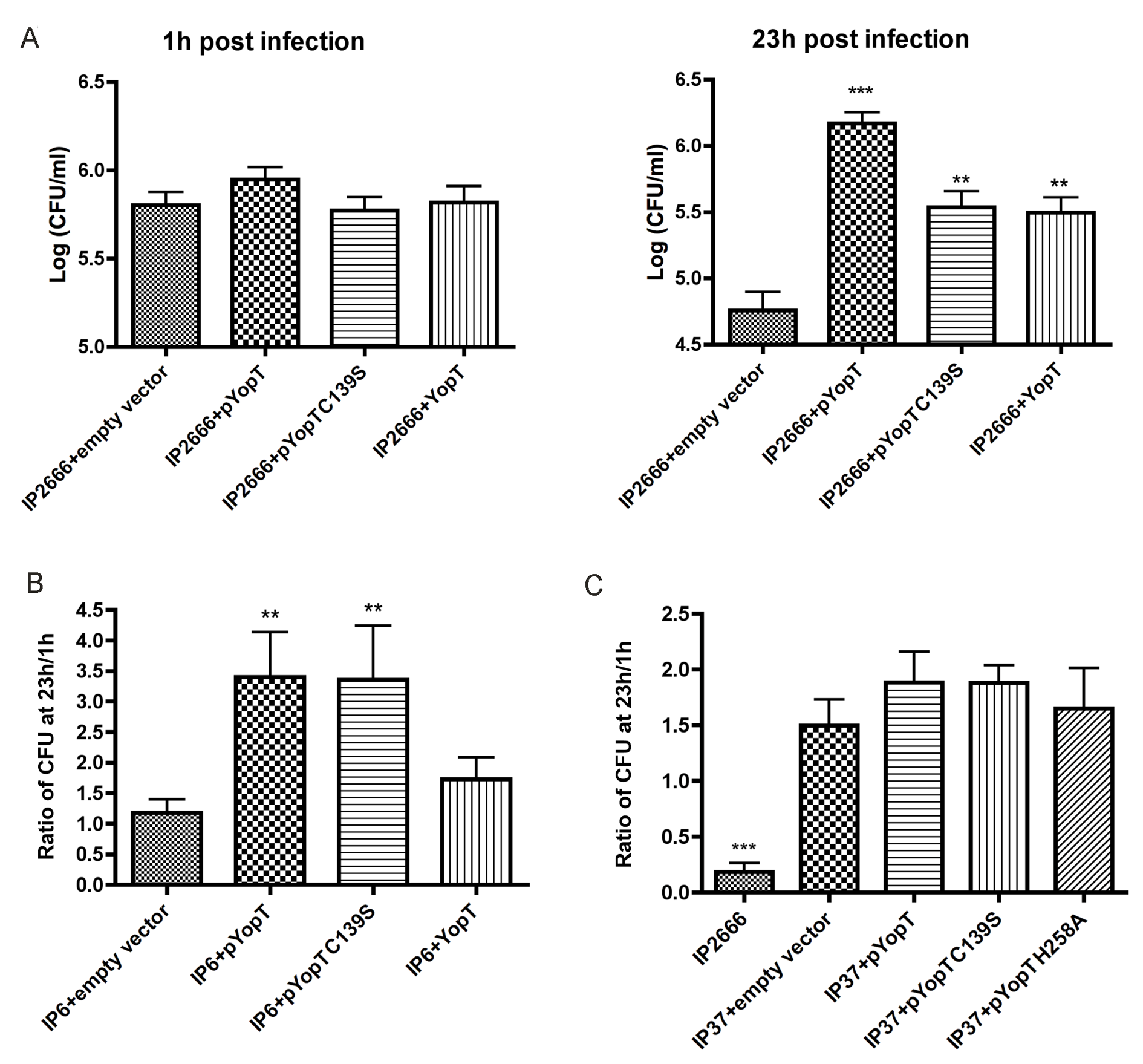

Supplement: Figure S6 — Survival of Y. pseudotuberculosis inside macrophages determined by CFU assay as described in Figure 1. (A) The logarithm of intracellular bacteria count per well at 1 h post infection and 23 h post infection. **, P<0.01 and ***, P<0.001 compared to IP2666+empty vector. (B) Ratio of CFU at 23 h/1 h. **, P<0.01 compared to IP6+empty vector. (C) Ratio of CFU at 23 h/1 h. ***, P<0.001 compared to IP37+empty vector. Results shown are the means from at least three independent experiments with duplicate infection wells. Error bars show standard deviations. (TIF) [file ppat.1004346.s006.tif]

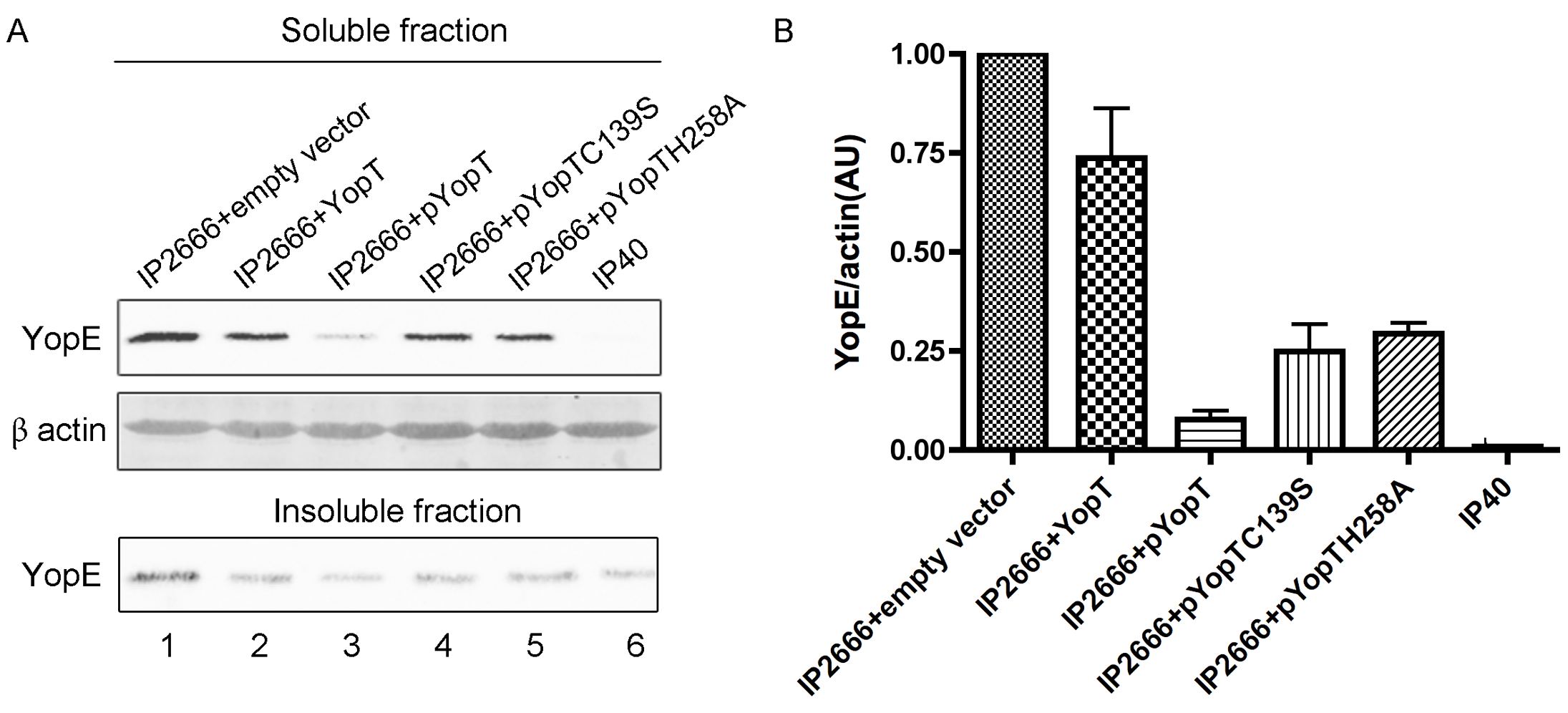

Supplement: Figure S7 — Measurement of YopE translocation in macrophages by different Y. pseudotuberculosis strains. (A) YopE translocation levels in BMDMs infected by the indicated strains, determined by detergent extraction assay and immunoblotting analysis using anti-YopE antibodies, as described in Figure 5. β-actin levels from the soluble fraction are shown as loading controls. (B) The intensity of each band was calculated using Odyssey IR imaging system, and the YopE/β actin ratios were normalized according to IP2666+empty vector. Results shown are the means from two independent experiments. Error bars show standard deviations. (TIF) [file ppat.1004346.s007.tif]

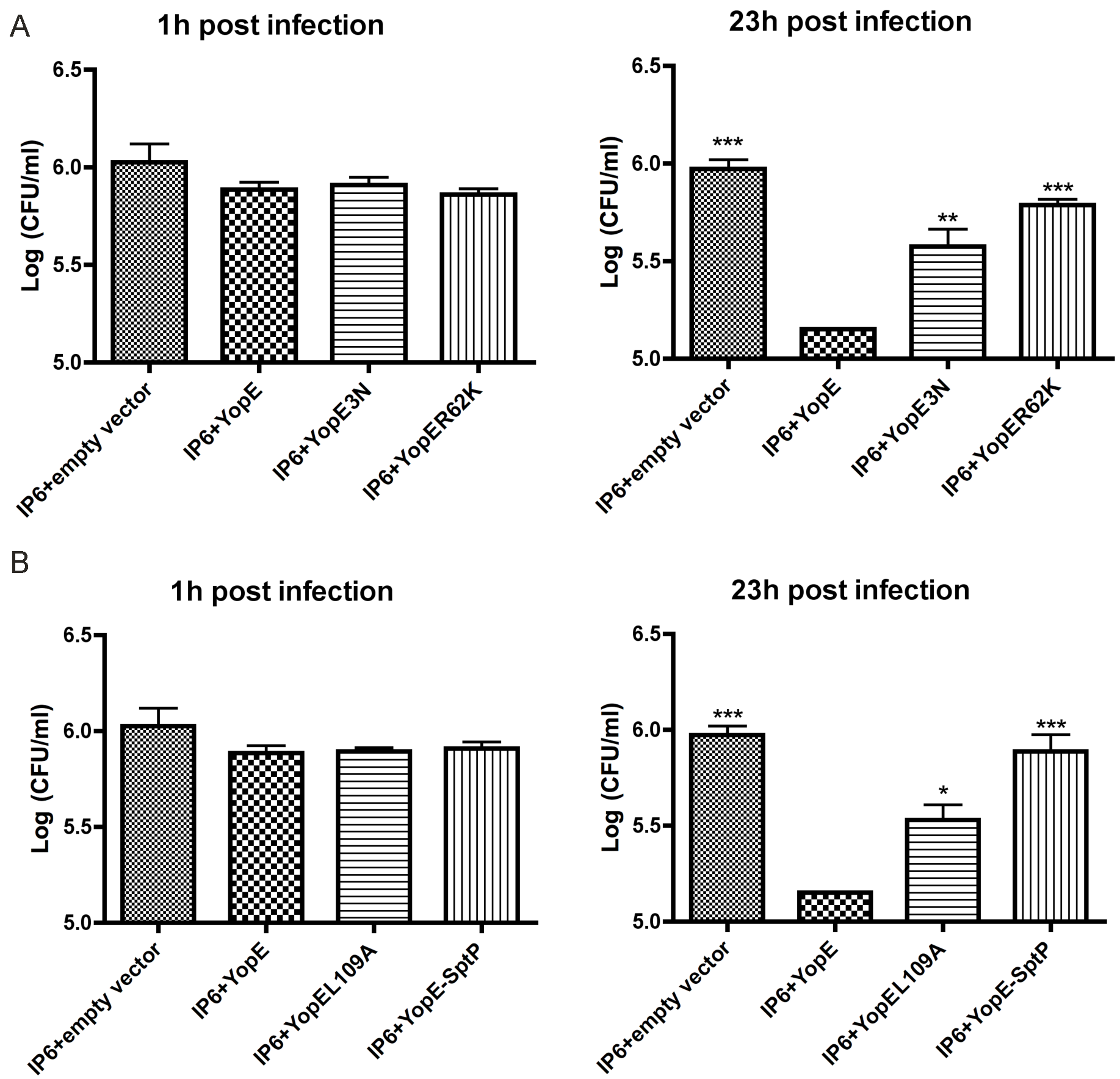

Supplement: Figure S8 — Survival of Y. pseudotuberculosis strains determined by CFU assay as described in Figure 1. Shown is the logarithm of intracellular bacteria count per well at 1 h post infection and 23 h post infection comparing IP6+empty vector, IP6+YopE, IP6+YopE3N and IP6+YopER62K (A) or IP6+empty vector, IP6+YopE, IP6+YopEL109A and IP6+YopE-SptP (B). Results are the means from three independent experiments with duplicate infection wells. Error bars show standard deviations. *, P<0.05; **, P<0.01 and ***, P<0.001 compared to IP6+YopE, as determined by one-way ANOVA. (TIF) [file ppat.1004346.s008.tif]

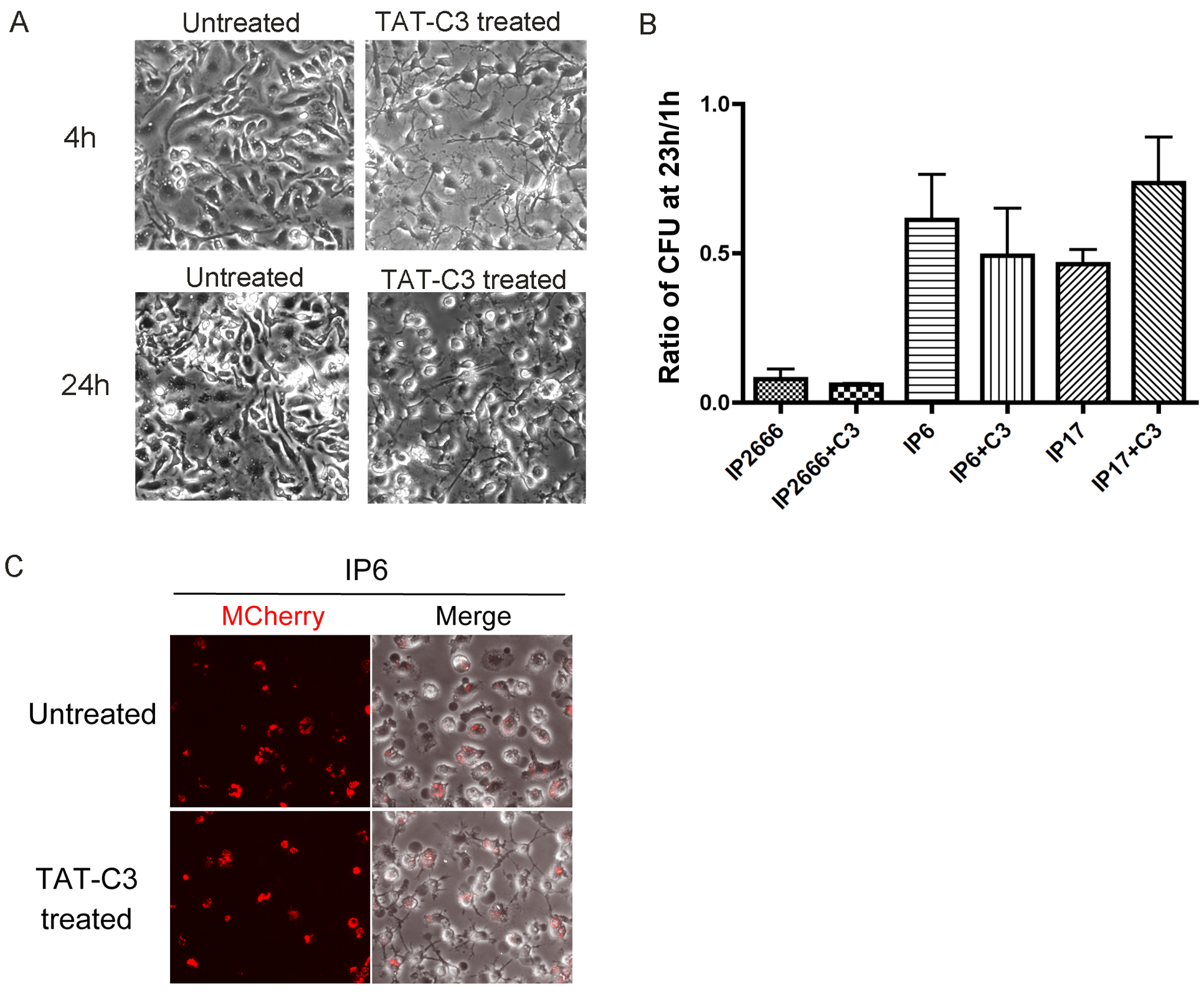

Supplement: Figure S9 — Survival of Y. pseudotuberculosis strains inside macrophages, in the presence or absence of TAT-C3. (A) Morphological changes of BMDMs upon TAT-C3 treatment was determined by phase contrast microscopy. Shown are BMDMs treated with 10 µg/ml TAT-C3 for 4 h or 24 h. BMDMs with no treatment are also shown as controls. (B) Intracellular bacterial survival was determined by CFU assay, as described in Figure 1. When indicated, 10 µg/ml TAT-C3 was present throughout the experiment. Results shown are the means from three independent experiments with duplicate infection wells. There is no significant difference in the survival of each strain with TAT-C3 treatment as compared individually to that without treatment. (C) Intracellular survival of mCherry encoding IP6 was determined by fluorescence microcopy, as described in Figure 7. When indicated, 10 µg/ml TAT-C3 was added throughout the experiment. Shown is mCherry or an overlay of mCherry and phase contrast signal at 24 h post infection. (TIF) [file ppat.1004346.s009.tif]
